# Impact of Cassia Bark Consumption on Glucose and Lipid Control in Type 2 Diabetes: An Updated Systematic Review and Meta-Analysis

**DOI:** 10.7759/cureus.16376

**Published:** 2021-07-13

**Authors:** Anindita Mandal, Suresh Sharma, Ritu Rani, Shashi Ranjan, Ravi Kant, Anissa Mirza

**Affiliations:** 1 Nursing, All India Institute of Medical Sciences, Rishikesh, IND; 2 Nursing, All India Institute of Medical Sciences, Jodhpur, IND; 3 Biochemistry, All India Institute of Medical Sciences, Rishikesh, IND; 4 General Medicine, All India Institute of Medical Sciences, Rishikesh, IND

**Keywords:** cinnamomum cassia, chinese cinnamon, types 2 diabetes, serum blood glucose, lipid profile

## Abstract

Control of diabetes is a constant challenge and natural remedies are being searched along with modern medicine. The effectiveness of cinnamon in managing it lacks consensus. Besides this, earlier trials had a variant in the type of product they used, quantity, duration, the form of molecules, etc. So, we aimed to measure the impact of cassia ground bark powder consumption, 1-2 gm/day for 90 days, in lowering plasma glucose and lipids among those with type 2 diabetes.

The authors searched the PubMed, Medline, Embase, CINAHL, Clinical Key, Ovid, and Scopus databases and the Cochrane Central Register (last search December 30, 2020) with the MeSH terms and keywords of cinnamon, cassia cinnamon, Chinese cinnamon, and type 2 diabetes mellitus to conclude the effects of cassia cinnamon on diabetes based on the evidence of human clinical trials that reported at least one of the following: glycosylated hemoglobin (HbA1C), fasting blood glucose, total cholesterol, triglycerides, low-density lipoprotein (LDL), and high-density lipoprotein (HDL).

Weighted mean differences were calculated by using the random-effect model of RevMan software (The Cochrane Collaboration, London, UK), and the pooled analysis found an insignificant reduction of the outcome variable (p>0.05).

## Introduction and background

Diabetes mellitus is an incurable metabolic disease, well-known as a leading cause of morbidity and mortality among the adult age group globally [1]. Strategies for managing hyperglycemia in diabetes mellitus include pharmacologic treatment, lifestyle modifications, and dietary changes [2]. The use of conventional hypoglycemic agents is a costlier treatment that may cause some of its side effects too, therefore natural, easy-going remedies are in demand. While plant resources become a chief target to search for new drugs, cinnamon or *dalchini* claimed an experimental spice for controlling diabetes. Cinnamon has come to be a natural product of interest, as it is an herb, with less price and negligible side effects, and has been hypothesized for its immense health benefits to lower serum lipids and blood glucose [3]. Cinnamaldehyde, an active molecule of cinnamon, has been preliminarily investigated for insulin release and regulation of insulin receptor kinase. Water-soluble polyphenol compounds extracted from cinnamon may enhance insulin sensitivity by inhibiting the enzyme protein tyrosine phosphatase 1B (PTPase 1B), which inactivates insulin receptors [4] and increases the glucose-transporting molecules (GLUT4) required for glucose uptake by adipose and muscle cells from plasma [5]. Though there are several randomized controlled trials (RCTs) where cinnamon has been studied for its glycemic and lipid-lowering effects, they ended up showing conflicting results. Even a prior meta-analysis was criticized because included studies were not uniform. There was very little similarity between studies regarding the use of a specific type of cinnamon, e.g. cinnamon zeylanicum vs. cassia; using part of it (leaves, bark, or root); the form of molecules, e.g. ground bark, water, or alcohol extract; the presence of an active compound, e.g. cinnamaldehyde or polyphenol; given doses, which varied from 0.5 to 6 gm; duration of treatment, e.g. one to three months; target population and sample size of experiments. Therefore, an updated meta-analysis of RCTs sharing similarities in the above factors was planned for evaluating a specific cinnamon effect (cassia cinnamon) on the glycemic and lipid profile among patients with type 2 diabetes mellitus. This review aims to conclude the question with quantitative synthesis from available scientific experimental data, revealed literature, and current evidence.

## Review

Search strategy

Using Preferred Reporting Items for Systematic Reviews and Meta-analysis (PRISMA) guidelines, review authors searched PubMed, Medline, Embase, Ovid, Discovery Search, Clinical Key, and the Cochrane Central Register (last search December 30, 2020) along with some hand search for finding studies eligible for this systematic review and meta-analysis. Keywords, free-text terms, and Mesh terms, such as “Cinnamomum cassia” OR “Chinese cinnamon” OR “Cassia Cinnamon”; “Cassia Cinnamon AND Serum blood glucose”; “Cassia Cinnamon AND Type 2 Diabetes Mellitus”; “Chinese cinnamon AND Anti-diabetic”; “Cinnamomum cassia AND Lipid profile”; “Chinese cinnamon AND serum blood cholesterol” were used for the purpose. Two reviewers searched individually and curtained potentially eligible studies via title, abstract, and related references to select literature that requires further detailed examination. Cross-references cited in retrieved articles were also reviewed to identify additional relevant studies. The discrepancy among the two reviewers has been resolved through discussion with the third independent reviewer. The present systematic review and meta-analysis have been registered in the International Prospective Register for Systematic Reviews (PROSPERO) and the reference ID is CRD42020183596.

Study selection

Studies that fulfilled the following criteria’s were selected and included: 1) studies examining the efficacy of cassia on type 2 diabetes among the adult age group (age more than 18 years) stated at least one of the following outcome variables: glycosylated hemoglobin (HbA1C), fasting blood glucose, total cholesterol, high-density-lipoprotein, low-density-lipoprotein, and triglycerides; 2) full-text articles available in the English language; 3) all experimental, comparative studies and clinical trials till the date of search. We let off papers or studies such as the following: 1) case reports, letters, editorials, opinions, commentaries, review papers, etc.; 2) studies on other properties or biological values of Cinnamomum (C.) cassia instead of its anti-diabetic and hypolipidemic role; 3) Studies that didn’t mention species of cinnamon they have used or if they had used an intervention other than cassia bark powder.

Data extraction

**Figure 1:**
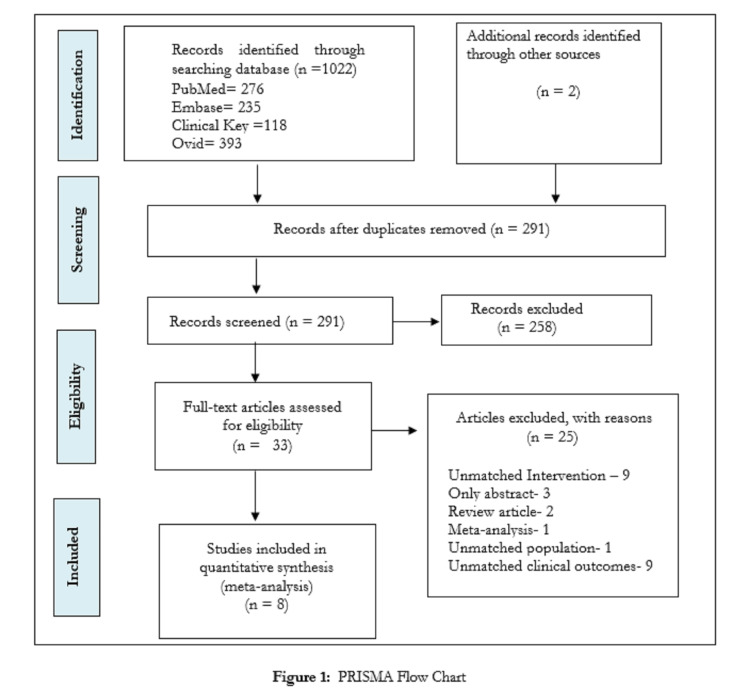
PRISMA flow chart PRISMA: Preferred Reporting Items for Systematic Reviews and Meta-Analysis

Review authors performed a literature search till December 30, 2020, as per the PRISMA guidelines (Figure 1) [6]. A total of 1022 studies were identified by using different databases (PubMed - 276, Embase - 235, Ovid - 393, Clinical Key - 118, respectively, while two studies were from a hand search and search from other databases). We assessed 33 studies for the eligibility criteria of our systematic review and finally included eight clinical trials for quantitative synthesis or meta-analysis. The reasons for the exclusion of 25 studies were: only published abstract/no full text - 3; unmatched intervention - 9 (use of cinnamon extract - 4, type of cinnamon was not specified - 3, use of cinnamon zeylanicum - 2); review article - 2; meta-analysis - 1; unmatched population - 1 (type 1 diabetes mellitus); and unmatched clinical outcome variable - 9. Primary reviewers took out the data from the included studies by using a data extraction form, and for further enhancement of the authenticity of the extracted data, another one cross-checked it all. In case of any queries related to the study findings, the corresponding author was contacted. The present meta-analysis included eight clinical trials that shared methodological similarities with each other and used cassia bark powder 1-2 gm per day in capsule form for 40 to 90 days among the type 2 diabetic adult population [7-14].

Methodological quality assessment

**Figure 2:**
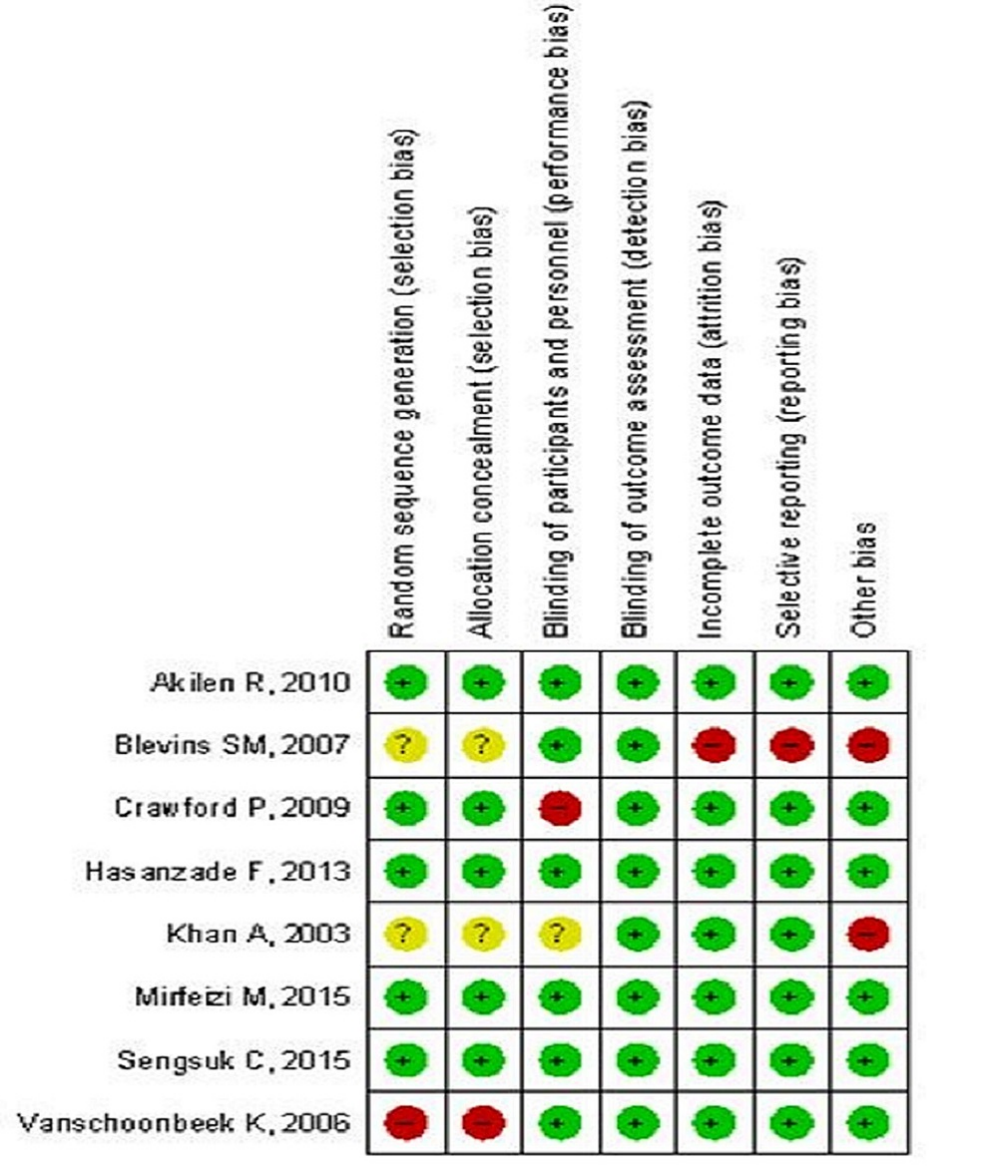
Risk-of-bias summary Review authors' judgment of each risk-of-bias item for each included study.

**Figure 3:**
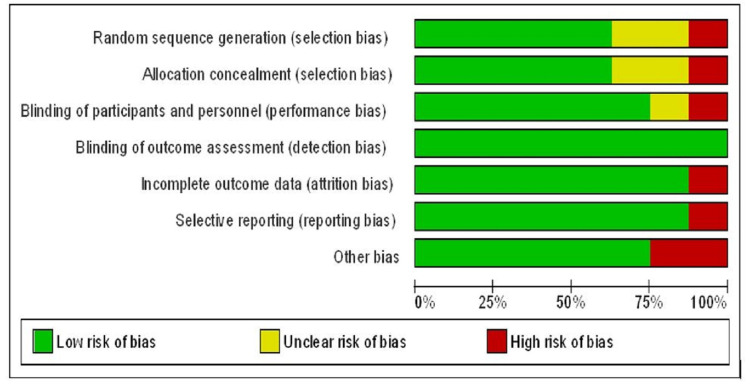
Risk-of-bias graph Review authors' judgment of each risk-of-bias item presented as percentages across all included studies.

Included trials were meticulously revised and evaluated for risk-of-bias by two reviewers independently using the Cochrane Collaboration approach or Cochrane risk-of-bias assessment tool [15]. This checklist included seven validity questions covering the following domains: random sequence generation, allocation concealment, blinding of participants and personnel, blinding of outcome assessment, incomplete outcome data, selective reporting, and other bias. All studies were described as low risk, high risk, and unclear risk on behalf of their bias toward each domain (Figure 2 and Figure 3). In case a study reported low risk for all domains, it was considered to be of good quality and vice-versa. If there is any conflict between primary reviewers regarding the risk-of-bias assessment, decisions were ended with mutual consensus after a thorough assessment by other reviewers. This review did not have a minimum number of trials required for the outcomes to provide a funnel plot, so no funnel plot was developed for reporting publication bias.

Abstraction of data

Subsequent information had been taken from every trial (Table 1): the identity of the author, year of publication, area of study, sample size, name of cinnamon species, cinnamon dose (including frequency), duration of treatment, and changes in parameters or outcome variable (glycosylated hemoglobin, plasma fasting glucose, triglyceride, LDL, HDL, and total cholesterol).

**Table 1:** Baseline characteristics, interventions, and outcomes of included trials BD - twice a day; TDS - three times daily; FBG - fasting blood glucose; HbA1C - glycosylated hemoglobin; HDL - high-density lipoprotein; LDL - low-density lipoprotein

Study (Author, Year, Area)	Participants (Type 2 diabetic adult people divided into groups)	Treatment/Control/Placebo	Duration	Outcome
Khan A, 2003, Pakistan [6]	Cinnamon group - 10; Placebo group - 10	C. cassia bark powder 500 mg capsule BD. Placebo - wheat flour 500 mg capsule BD.	40 days	Significant (p<0.05) reduction had been seen in the level of fasting blood glucose, triglyceride, total cholesterol, and LDL cholesterol. Non-significant (p>0.05) changes were found in the HDL cholesterol level.
Vanschoonbeek K, 2006, Netherlands [7]	Cinnamon group - 12; Placebo group - 13	C. cassia bark powder 500 mg capsule TDS. Placebo - not specified 500 mg capsule TDS.	6 weeks	There was no time× treatment interaction for fasting blood glucose, HbA1C, triglycerides, total cholesterol, and LDL and HDL cholesterol (p>0.05).
Blevins SM, 2007, USA [8]	Cinnamon group - 29; Placebo group - 28. Intention to treat analysis was used.	C. cassia bark powder 500 mg capsule BD. Placebo - wheat flour 500 mg capsule BD.	90 days/3 months	Non- significant results had been found (significance was set at 0.05). Values of outcome variable were FBG (p= 0.38); HbA1C (p= 0.64); triglyceride (p= 0.78); total cholesterol (p= 0.63); LDL (p=0.87); HDL (p= 0.28).
Crawford P, 2009, USA [9]	Cinnamon group - 55; Placebo group - 54. Intention to treat analysis was used.	Usual care with C. cassia bark powder capsules 500 mg BD. Control - Usual care alone.	90 days	There was a significant decrease of HbA1C (p<0.04) among the intervention group.
Akilen R, 2010, UK [10]	Cinnamon group - 30; Placebo group - 28. Intention to treat analysis was used.	C. cassia bark powder 500 mg capsule four times per day. Placebo - Starch powder 500 mg capsule 4 times per day.	12 weeks	Intake of cinnamon significantly reduced HbA1C (p= 0.029) when comparing the post-intervention mean of cinnamon and placebo group. The changes in fasting plasma glucose and lipid profiles were not significantly different from placebo (p>0.05).
Hasanzade F, 2013, Iran [11]	Cinnamon group - 35; Placebo group - 35.	C. cassia bark powder 500 mg capsule BD. Placebo - not specified 500 mg capsule BD.	60 days	There was no significant change in the level of FBG and HbA1C between the two groups after the intervention (P > 0.05).
Mirfeizi M, 2015, Iran [12]	Cinnamon group - 27; Placebo group - 45.	C. cassia bark powder 500 mg capsule BD. Placebo - starch 500 mg capsule BD.	90 days/3 month	Non- significant changes was found in the level of FBG (p=0.172); HbA1C (p=0.284); triglyceride (p=0.168); total cholesterol (p=0.965); and HDL (p=0.885), and the level of LDL was significantly reduced (p=0.048).
Sengsuk C, 2015, Thailand [13]	Cinnamon group - 49; Placebo group - 50.	C. cassia bark powder 500 mg capsule TDS. Placebo - not specified 500 mg capsule TDS.	60 days	There was a significant reduction in the level of FBG, HbA1c, and triglyceride, and an increase of HDL (p<0.001). No desirable change had been found in LDL and total cholesterol (p>.001).

Statistical analysis

There was variation in the unit of outcome variables among eight included studies (mmol vs. mg/dl). Therefore, to create a uniform digit, all parameters had been converted into mg/dl. The mean changes in fasting blood glucose, HbA1C, and lipid parameters from the baseline remained as continuous variables; the mean differences (MDs) were pooled and entered into generic inverse variance, RevMan 5.4 (The Cochrane Collaboration, London, UK), to conduct a meta-analysis using its random-effects model. In case mean differences between the baseline and end of the study were not reported directly, they were calculated by using statistical software Comprehensive Meta-Analysis Software (CMA) version 3 trial (Biostat, Englewood, NJ). The trial by Khan et al. evaluated three altered doses of C. cassia and compared each with its parallel placebo [7] but we have considered one dose among three (1 gm. per day) as per the present manuscript inclusion criteria. A p-value of <0.05 was set as statistically significant. The I^2^ statistic measured the statistical heterogeneity; values of 75%, 50%, and 25%, respectively, denote high, medium, and low degrees of heterogeneity, though low levels are desired.

Results

Study Characteristics

Next to the screening, eight clinical trials (n=510 participants) were considered for the present systematic review and meta-analysis (Figure 1). Seven trials out of eight reported usable data for fasting blood glucose (except Crawford P) [10] and glycosylated hemoglobin level (except Khan A) [7]. Lipid parameters (triglyceride, total cholesterol, LDL-C, HDL-C) had been reported by six trials, except Hasanzade F et al. [12] and Crawford P [10]. But during the analysis of HDL, only four trials had been involved because there was a mistake in putting the value of HDL in the studies done by Khan et al. [7] and Blevins SM [9]. Three studies [9-11] had specified loss to follow up and intention to treat analysis. All the trials detailed the administration of cinnamon relating to food.

Quantitative Synthesis

**Figure 4:**
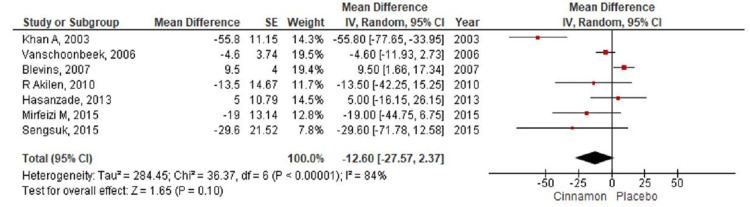
Forest plot showing effects of cinnamon on fasting blood glucose CI - confidence interval; SE - standard error

**Figure 5:**
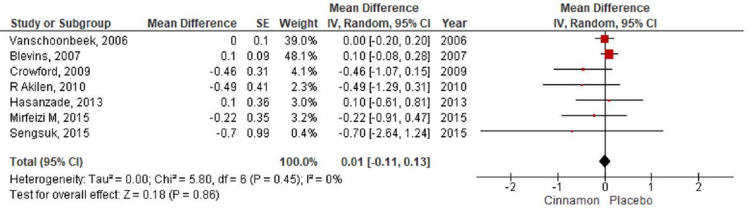
Forest plot showing the effects of cinnamon on glycosylated hemoglobin CI - confidence interval; SE - standard error

**Figure 6:**
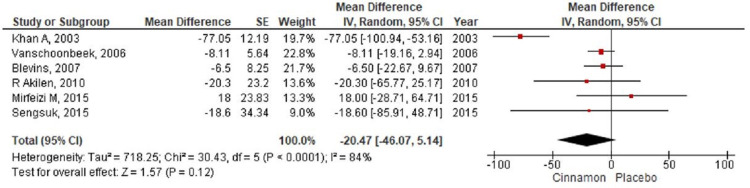
Forest plot showing the effects of cinnamon on serum triglycerides CI - confidence interval; SE - standard error

**Figure 7:**
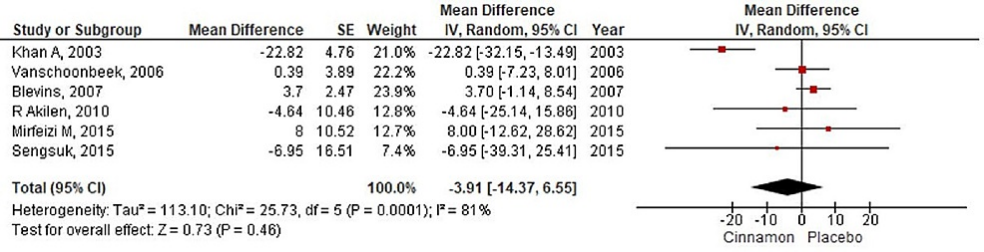
Forest plot showing the effects of cinnamon on total cholesterol CI - confidence interval; SE - standard error

**Figure 8:**
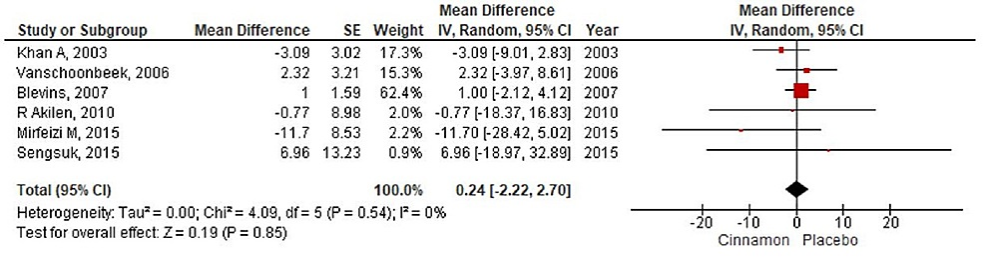
Forest plot showing the effects of cinnamon on LDL cholesterol CI - confidence interval; SE - standard error; LDL - low-density lipoprotein

**Figure 9:**
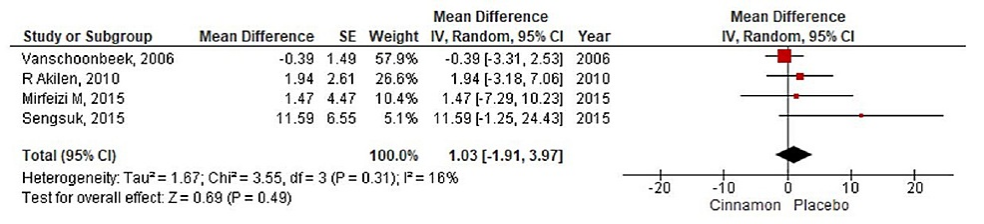
Forest plot showing the effects of cinnamon on HDL cholesterol CI - confidence interval; SE - standard error; HDL - high-density lipoprotein

In the present meta-analysis, seven studies [7-9,11-14] were identified that reported levels of fasting blood glucose with 401 patients randomized for cinnamon and placebo group (Cinnamon = 192; Placebo = 209). The pooling of results demonstrated a reduction in the fasting blood glucose level (MD-12.60; 95% CI: -27.57 to 2.37; P=0.1) in the cinnamon group as compared to the placebo group; however, these findings were not statistically significant (Figure 4). The HbA1c level was reported by seven RCTs [8-14] with 490 participants (Cinnamon = 237; Placebo = 253) and a pooled analysis of the parameters revealed no significant changes in the level of HbA1C in the cinnamon group as compared to the placebo group (MD 0.01; 95% CI: -0.11 to 0.13; P=0.86) (Figure 5). Six studies [7-9,11,13-14] measured the differences in triglyceride level, total cholesterol level, and LDL cholesterol level between the cinnamon and placebo group with 331 participants (Cinnamon = 157; Placebo = 174). Analysis reported no statistically significant reduction in the level of triglyceride (MD-20.47; 95% CI: -46.07 to 5.14; P=0.12) (Figure 6) and total cholesterol (MD-3.91; 95% CI: -14.37 to 6.55; P=0.46) (Figure 7) and showed negligible changes in the level of LDL cholesterol (MD 0.24; 95% CI: -2.22 to 2.70; P=0.85) (Figure 8). No significant increase in the effect of cinnamon compared with placebo on HDL cholesterol (MD 1.03; 95% CI: -1.91 to 3.97; P=0.49) was identified by four clinical trials [8,11,13-14] with a total of 254 participants (Cinnamon = 118; Placebo = 136) (Figure 9). A variant degree of heterogeneity was present between the studies while analyzing all outcome variables (I^2^ ranging from 0% to 84%).

Discussion

Type 2 diabetes mellitus is the most common, serious, long-term ailment, with a major impact on the lives and well-being of individuals, families, and society [16]. It is amid the topmost causes of demise in adults, which affects a significant percentage of the population (estimated to be 9.3%, 2019) throughout the world [1]. Cinnamon is a common food spice and a natural health product (NHP) [17], which was claimed as antidiabetic from ancient times, but the species and doses remain unclear. As a source of cinnamon spice, various species are cultivated, e.g. Ceylon cinnamon or C. zeylanicum; Chinese cinnamon or C. cassia; Vietnamese cinnamon or C. loureiroi; Malabar cinnamon or C. tamala; Indonesian cinnamon or C. burmannii; and Camphor laurel or C. camphora [18].

Cinnamon has been of research interest in patients with diabetes since the 1990s when peroxisome proliferator-activated receptors (PPARs) became recognized as a possible therapeutic target for dyslipidemia and diabetes [4]. In the field of molecular biology, PPARs are a group of nuclear receptor proteins that function as transcription factors regulating the expression of genes. In well-controlled laboratory settings, studies had supported that cinnamon extract significantly inhibited messenger RNA (mRNA) expression of genes of the inflammatory cytokine interleukin-6, interleukin-1β, and tumor necrosis factor-α. The cinnamon extract also augmented the mRNA expression of genes, which leads to increased insulin sensitivity, e.g. Ir, Irs-1, Irs-2, PI-3 kinase, along with Akt1, and repressed genes related to an increase of triacylglycerols, cholesterol, and levels of apolipoprotein-B48, etc. The stimulation of phospho-p38 mitogen-activated protein kinase, c-Jun N-terminal kinase, and extracellular-signal-regulated kinase expressions also had been demonstrated by cinnamon extract in laboratory experiments [19]. Sriramajayam K et al. confirmed that CE ameliorates type 2 diabetes by inducing glucose transporter 4 translocation via suppressed AMPK signaling pathway in adipocyte, which further helps in glucose uptake [20]. Cao H et al. stated polyphenols are one of the active anti-diabetic compounds of cinnamon extract that influence the expression of the insulin receptor, tristetraprolin, and GLUT4 [21]. Babu SP et al. verified cinnamaldehyde-A as a potential anti-diabetic agent as the oral administration of cinnamaldehyde (20 mg/kg bwt) significantly decreased plasma glucose concentration, glycosylated hemoglobin (HbA1C), serum total cholesterol, triglyceride levels, and, at the same time, markedly increased plasma insulin, hepatic glycogen, and high-density lipoprotein-cholesterol levels in an animal study. Also, cinnamaldehyde restored the altered plasma enzyme (aspartate aminotransferase, alanine aminotransferase, lactate dehydrogenase, alkaline phosphatase, and acid phosphatase) levels to near normal [22].

But when it comes to a clinical study, both meta-analyses and RCTs have shown very conflicting results. Davis PA et al. found cinnamon intake, either as whole cinnamon or as a cinnamon extract, was statistically significant in lowering FBG (-0.49±0.2 mmol/L; n=8, P=.025) in their comprehensive meta-analysis (Biostat Inc.) in the year 2011 [23]. But Leach MJ et al. [24] and Baker et al. [25] found cinnamon to be no more effective than placebo, another active medication, or no treatment in reducing glucose levels and glycosylated hemoglobin A1C (HbA1c) in their systematic review. Any absolute decisions can’t be drawn about the use of cinnamon as an anti-diabetic therapy by Kirkham S et al. [26].

Among RCTs also, contradictory results have been found. Some researchers had worked on an aqueous extract of cinnamon and found it effective in improving glycemic control in type 2 diabetes though they were not included in the present systematic review, as it had been performed on whole cinnamon or cinnamon bark powder [27-30]. Some studies, other than the included eight trials, used cinnamon bark and concluded significant efficacy in managing type 2 diabetes but cannot be included because of not mentioning its specific species [31-33] or non-availability of full text [34]. Non-efficacy of cinnamon on type 2 diabetes had been shown in some studies: Suppapitiporn S. et al. [35] used cinnamon cassia (though the study was excluded because of dose difference), Vafa M. et al. [36] used cinnamon zeylanicum, and Justin A et al. [37] included type 1 diabetic patients. Authors have suggested some extraneous variables in clinical studies, which are the reasons for conflicting results. For example, the number of active molecules (cinnamaldehyde or polyphenols) may differ among various species and even among formulations [38]. Furthermore, dissimilarities in manufacturing practices might affect the number of ingredients found in a specific formulation because herbal medicine doesn’t usually undergo the same manufacturing oversights as other pharmacy products. Other more definitive reasons are not following dietary restrictions, lifestyle modifications, and drug adherence by study participants in included trials. Variation in food habits, exercise, interactions with different pharmacotherapy, and poor drug compliance manipulate the results of human clinical trials.

## Conclusions

The present meta-analysis concludes that the ingestion of powdered cassia (1-2 gm) doesn’t decrease fasting blood glucose, glycosylated hemoglobin, total cholesterol, triglycerides, and low-density lipoprotein and has no effects on the significant increase in high-density lipoprotein among patients with type 2 diabetes. As included studies in the present meta-analysis had a small sample size, it is recommended to conduct multi-centric trials with a well-planned, robust methodology, large sample size, long duration, and follow-up. The ingestion of a high dose (at least 3 gm) of cassia bark powder or cassia extract alone (without other medicines or complementary therapy) along with the strict implementation of a lifestyle and diet protocol would be more effective to evaluate its outcomes on glucose and lipid control in type 2 diabetes mellitus. A clinical trial on cinnamon zeylanicum is also recommended.

## References

[1] Saeedi P, Petersohn I, Salpea P (2019). Global and regional diabetes prevalence estimates for 2019 and projections for 2030 and 2045: results from the International Diabetes Federation Diabetes Atlas, 9th edition. Diabetes Res Clin Pract.

[2] Allen RW, Schwartzman E, Baker WL, Coleman CI, Phung OJ (2013). Cinnamon use in type 2 diabetes: an updated systematic review and meta-analysis. Ann Fam Med.

[3] Ulbricht C, Seamon E, Windsor RC (2011). An evidence-based systematic review of cinnamon (Cinnamomum spp.) by the Natural Standard Research Collaboration. J Diet Suppl.

[4] Sheng X, Zhang Y, Gong Z, Huang C, Zang YQ (2008). Improved insulin resistance and lipid metabolism by cinnamon extract through activation of peroxisome proliferator-activated receptors. PPAR Res.

[5] Anand P, Murali KY, Tandon V, Murthy PS, Chandra R (2010). Insulinotropic effect of cinnamaldehyde on transcriptional regulation of pyruvate kinase, phosphoenolpyruvate carboxykinase, and GLUT4 translocation in experimental diabetic rats. Chem Biol Interact.

[6] Moher D, Liberati A, Tetzlaff J, Altman DG, and the PRISMA Group (2009). Preferred reporting items for systematic reviews and meta-analyses: the PRISMA statement. PLoS Med.

[7] Khan A, Safdar M, Ali Khan MM, Khattak KN, Anderson RA (2003). Cinnamon improves glucose and lipids of people with type 2 diabetes. Diabetes Care.

[8] Vanschoonbeek K, Thomassen BJ, Senden JM, Wodzig WK, van Loon LJ (2006). Cinnamon supplementation does not improve glycemic control in postmenopausal type 2 diabetes patients. J Nutr.

[9] Blevins SM, Leyva MJ, Brown J, Wright J, Scofield RH, Aston CE (2007). Effect of cinnamon on glucose and lipid levels in non insulin-dependent type 2 diabetes. Diabetes Care.

[10] Crawford P (2009). Effectiveness of cinnamon for lowering hemoglobin A1C in patients with type 2 diabetes: a randomized, controlled trial. J Am Board Fam Med.

[11] Akilen R, Tsiami A, Devendra D, Robinson N (2010). Glycated haemoglobin and blood pressure-lowering effect of cinnamon in multi-ethnic type 2 diabetic patients in the UK: a randomized, placebo-controlled, double-blind clinical trial. Diabet Med.

[12] Hasanzade F, Toliat M, Emami SA, Emamimoghaadam Z (2013). The effect of cinnamon on glucose of type II diabetes patients. J Tradit Complement Med.

[13] Mirfeizi M, Mehdizadeh Tourzani Z, Mirfeizi SZ, Asghari Jafarabadi M, Rezvani HR, Afzali M (2016). Controlling type 2 diabetes mellitus with herbal medicines: a triple-blind randomized clinical trial of efficacy and safety. J Diabetes.

[14] Sengsuk C, Sanguanwong S, Tangvarasittichai O, Tangvarasittichai S (2016). Effect of cinnamon supplementation on glucose, lipids levels, glomerular filtration rate, and blood pressure of subjects with type 2 diabetes mellitus. Diabetol Int.

[15] Higgins JP, Altman DG, Gøtzsche PC (2011). The Cochrane Collaboration's tool for assessing risk of bias in randomised trials. BMJ.

[16] Trikkalinou A, Papazafiropoulou AK, Melidonis A (2017). Type 2 diabetes and quality of life. World J Diabetes.

[17] Nahas R, Goguen J (2013). Natural health products. Can J Diabetes.

[18] Sharma S, Mandal A, Kant R, Jachak S, Jagzape M (2020). Is cinnamon efficacious for glycemic control in type-2 diabetes mellitus. J Pak Med Assoc.

[19] Qin B, Dawson HD, Schoene NW, Polansky MM, Anderson RA (2012). Cinnamon polyphenols regulate multiple metabolic pathways involved in insulin signaling and intestinal lipoprotein metabolism of small intestinal enterocytes. Nutrition.

[20] Kannappan S, Jayaraman T, Rajasekar P, Ravichandran MK, Anuradha CV (2006). Cinnamon bark extract improves glucose metabolism and lipid profile in the fructose-fed rat, an animal model of insulin resistance. Singapore Med J.

[21] Cao H, Polansky MM, Anderson RA (2007). Cinnamon extract and polyphenols affect the expression of tristetraprolin, insulin receptor, and glucose transporter 4 in mouse 3T3-L1 adipocytes. Arch Biochem Biophys.

[22] Subash Babu P, Prabuseenivasan S, Ignacimuthu S (2007). Cinnamaldehyde—a potential antidiabetic agent. Phytomedicine.

[23] Davis PA, Yokoyama W (2011). Cinnamon intake lowers fasting blood glucose: meta-analysis. J Med Food.

[24] Leach MJ, Kumar S (2012). Cinnamon for diabetes mellitus. Cochrane Database Syst Rev.

[25] Baker WL, Gutierrez-Williams G, White CM, Kluger J, Coleman CI (2008). Effect of cinnamon on glucose control and lipid parameters. Diabetes Care.

[26] Kirkham S, Akilen R, Sharma S, Tsiami A (2009). The potential of cinnamon to reduce blood glucose levels in patients with type 2 diabetes and insulin resistance. Diabetes Obes Metab.

[27] Mang B, Wolters M, Schmitt B, Kelb K, Lichtinghagen R, Stichtenoth DO, Hahn A (2006). Effects of a cinnamon extract on plasma glucose, HbA, and serum lipids in diabetes mellitus type 2. Eur J Clin Invest.

[28] Stoecker BJ, Zhan Z, Luo R (2010). Cinnamon extract lowers blood glucose in hyperglycaemic subjects. FASEB J.

[29] Lu T, Sheng H, Wu J, Cheng Y, Zhu J, Chen Y (2012). Cinnamon extract improves fasting blood glucose and glycosylated hemoglobin level in Chinese patients with type 2 diabetes. Nutr Res.

[30] Anderson RA, Zhan Z, Luo R (2016). Cinnamon extract lowers glucose, insulin and cholesterol in people with elevated serum glucose. J Tradit Complement Med.

[31] Khan R, Khan Z, Shah S (2010). Cinnamon may reduce glucose, lipid and cholesterol level in type 2 diabetic individuals. Pakistan J Nutr.

[32] Sharma P, Sharma S, Agrawal RP, Agrawal V, Singhal S (2012). A randomized double blind placebo control trial of cinnamon supplementation on glycemic control and lipid profile in type 2 diabetes mellitus. Aust J Herbal Med.

[33] Al-Yasiry K, Kathum W, Alganimi YK, Nizar Z, Ewadh M (2014). Evaluation of anti-diabetic effect of cinnamon in patients with diabetes mellitus type II in Kerbala City. Nat Sci Res.

[34] Soni R, Bhatnagar V (2009). Effect of cinnamon (Cinnamomum cassia) intervention on blood glucose of middle aged adult male with non-insulin dependent diabetes mellitus (NIDDM). Ethno-Med.

[35] Suppapitiporn S, Kanpaksi N, Suppapitiporn S (2006). The effect of cinnamon cassia powder in type 2 diabetes mellitus. J Med Assoc Thai.

[36] Vafa M, Mohammadi F, Shidfar F (2012). Effects of cinnamon consumption on glycemic status, lipid profile and body composition in type 2 diabetic patients. Int J Prev Med.

[37] Altschuler JA, Casella SJ, MacKenzie TA, Curtis KM (2007). The effect of cinnamon on A1C among adolescents with type 1 diabetes. Diabetes Care.

[38] Corns CM (2003). Herbal remedies and clinical biochemistry. Ann Clin Biochem.

